# Membrane Perturbation of ADP-insensitive Phosphoenzyme of Ca^2+^-ATPase Modifies Gathering of Transmembrane Helix M2 with Cytoplasmic Domains and Luminal Gating

**DOI:** 10.1038/srep41172

**Published:** 2017-01-24

**Authors:** Stefania Danko, Kazuo Yamasaki, Takashi Daiho, Hiroshi Suzuki

**Affiliations:** 1 Asahikawa Medical University, Department of Biochemistry, Midorigaoka-Higashi, Asahikawa, 078-8510, Japan

## Abstract

Ca^2+^ transport by sarcoplasmic reticulum Ca^2+^-ATPase involves ATP-dependent phosphorylation of a catalytic aspartic acid residue. The key process, luminal Ca^2+^ release occurs upon phosphoenzyme isomerization, abbreviated as *E*1PCa_2_ (reactive to ADP regenerating ATP and with two occluded Ca^2+^ at transport sites) → *E*2P (insensitive to ADP and after Ca^2+^ release). The isomerization involves gathering of cytoplasmic actuator and phosphorylation domains with second transmembrane helix (M2), and is epitomized by protection of a Leu^119^-proteinase K (prtK) cleavage site on M2. Ca^2+^ binding to the luminal transport sites of *E*2P, producing *E*2PCa_2_ before Ca^2+^-release exposes the prtK-site. Here we explore *E*2P structure to further elucidate luminal gating mechanism and effect of membrane perturbation. We find that ground state *E*2P becomes cleavable at Leu^119^ in a non-solubilizing concentration of detergent C_12_E_8_ at pH 7.4, indicating a shift towards a more *E*2PCa_2_-like state. Cleavage is accelerated by Mg^2+^ binding to luminal transport sites and blocked by their protonation at pH 6.0. Results indicate that possible disruption of phospholipid-protein interactions strongly favors an *E*2P species with looser head domain interactions at M2 and responsive to specific ligand binding at the transport sites, likely an early flexible intermediate in the development towards ground state *E*2P.

Sarco(endo)plasmic reticulum (SR) Ca^2+^-ATPase (expressed in adult fast-twitch skeletal muscle, SERCA1a), a representative member of P-type ion transporting ATPases, catalyzes Ca^2+^ transport coupled with ATP hydrolysis (Fig. 1) (for recent reviews, see Refs 1, 2, 3). The enzyme consists of three large cytoplasmic domains, Nucleotide binding (N), Phosphorylation (P), and Actuator (A), and ten transmembrane helices (M1~M10) (Figs 1 and 2). Ca^2+^ transport requires communication between the catalytic site on the cytoplasmic domains and the transport sites in the transmembrane helices *via* coupled structural changes, *i.e.* cytoplasmic domain motions and rearrangements of transmembrane helices. The enzyme is activated by the binding of two cytoplasmic Ca^2+^ ions at the high affinity transport sites composed of residues located on M4, M5, M6, and M8 (*E*2 to *E*1Ca_2_ in Fig. 1). Then it is auto-phosphorylated at the catalytic residue Asp^351^ with ATP to form an ADP-sensitive phosphoenzyme (*E*1P), which is capable of reacting with ADP to regenerate ATP in the reverse reaction. Upon *E*1P formation, the two bound Ca^2+^ are occluded in the transport sites (*E*1PCa_2_). The subsequent isomeric transition to the ADP-insensitive *E*2P form involves a large rotation of the A domain to associate with the P domain, thereby rearranging the Ca^2+^ binding sites to deocclude Ca^2+^, open the release path (luminal gate), and reduce the affinity, thus allowing Ca^2+^ release into the lumen. As a consequence, the catalytic site in *E*2P is prepared for subsequent aspartyl phosphate hydrolysis by tightening of associated A and P domains. In the first step towards hydrolysis, progressing from the ground state to the transition state, namely *E*2P + H_2_O → *E*2~P^‡^, the transport sites are protonated and the luminal gate closes tightly, preventing luminal Ca^2+^ access and driving the process forward^4^,^5^. The cytoplasmic part of the second transmembrane helix, M2, plays a crucial role in coupling A-domain motion and tilting of the P domain during the rearrangements of transport sites^4^,^6^,^7^,^8^,^9^.

The *E*2P ground state, transition state (*E*2~P^‡^), and product complex (*E*2·P_i_) in the *E*2P hydrolysis process are mimicked by the stable structural analogs *E*2·BeF_3_^−^, *E*2·AlF_4_^−^, and *E*2·MgF_4_^2−^, respectively, as produced with the respective phosphate analogs for different configurational states^4^. Their crystal structures, without or with the potent inhibitor thapsigargin (TG), have been solved at atomic level^7^,^8^,^10^,^11^ following purification of the protein using a non-ionic detergent octaethylene glycol monododecyl ether (C_12_E_8_). Commensurate with the structural changes mentioned, the crystal structures are subtly different although the overall molecular structure of the compactly organized cytoplasmic A, P, and N domains with tightly bound BeF_3_^−^ and occluded Mg^2+^ at the catalytic site and the arrangement of transmembrane helices are similar. Namely, the *E*2·BeF_3_^−^ crystal produced at pH 7.0 in 50 mM Mg^2+^ has wide open transport sites (luminal gate open) with one bound Mg^2+ ^11 and that at pH 5.7, where the transport sites are protonated and Mg^2+^ is absent, the luminal access pathway is less open^8^ (Fig. 2). The structures with bound TG at a cavity surrounded by M3, M5, and M7, namely *E*2·BeF_3_^−^(TG), and those of *E*2·AlF_4_^−^(TG) and *E*2·MgF_4_^2−^(TG), are different again, and the luminal gate is tightly closed. The closure is associated with formation of hydrophobic interaction network, the Tyr^122^-hydrophobic cluster (Y122-HC) by Leu^119^/Tyr^122^ on the cytoplasmic part of M2 and five residues of the gathered A and P domains (Ile^179^/Leu^180^ (A), Val^705^/Val^726^ (P)) and A/M3-linker (Ile^232^ on the loop connecting the A domain with M3). Significantly, in the *E*2·BeF_3_^−^ crystals without TG, where the gate is open, the side chains of Leu^119^/Tyr^122^ are close but pointing away from the other gathered five residues, indicative of weaker domain interactions here (Fig. 2).

Extensive mutation and kinetic studies have demonstrated^12^,^13^,^14^,^15^ that all seven residues involved in Y122-HC including Leu^119^/Tyr^122^ are crucial for opening the gate, reducing Ca^2+^ affinity, and allowing rapid Ca^2+^-release (*E*2PCa_2_ → *E*2P + 2Ca^2+^), and for subsequent gate-closure and the formation of a catalytic site with hydrolytic ability. Investigation of the structural changes during these events has been aided by proteolytic digestion patterns, including a prtK site at Leu^119^ 4,16,17. The site is exposed in the unphosphorylated *E*2 form but protected in *E*2·BeF_3_^−^, *E*2·AlF_4_^−^, and *E*2·MgF_4_^2−^ as well as in the TG-bound forms of these analogs. Thus susceptibility to prtK attack or otherwise seems a good indicator of the state of the gathering of the head domains on M2. Significantly, *E*2PCa_2_ an early *E*2P species, is uniquely susceptible to attack, an indication of a loose arrangement of head domains on M2 prior to progression to ground state *E*2P^4^,^18^,^19^.

Unexpectedly, we now find that low, non-solubilizing concentrations of C_12_E_8_ render the prtK site at Leu^119^ in *E*2P (*E*2·BeF_3_^−^) susceptible to attack. It is as though the detergent has released constraints at the transmembrane helices to favor a state closer to that on Ca^2+^ binding to the luminal sites, namely *E*2PCa_2_. The phenomenon uncovers a hitherto undescribed intermediate just prior to ground state *E*2P, stabilized by detergent that is uniquely susceptible to diverse ligand binding and cross-protein conformational changes. It shows that phospholipid-protein interactions directly participate the conformational changes associated with luminal gating events and expedite Ca^2+^ release.

## Results

### PrtK-cleavage of Leu^119^-site in *E*2·BeF_3_^−^ with C_12_E_8_ at pH 7.4

In Fig. 3a, prtK-proteolysis of *E*2·BeF_3_^−^ is performed at pH 7.4 in 0.1 M K^+^ without and with a non-solubilizing low concentration of C_12_E_8_. In the absence of C_12_E_8_, *E*2·BeF_3_^−^ is completely resistant to prtK both without and with A23187 as found previously^4^. In the presence of C_12_E_8_, a 95-kDa fragment (p95) is produced by specific prtK-cleavage at the Leu^119^-site without any other cleavages. Cleavage is accelerated by 30 mM Mg^2+^, but no cleavage occurs in the absence of C_12_E_8_ even at 30 mM Mg^2+^. In Fig. 3d, the Mg^2+^ concentration dependence of the specific prtK-cleavage rate at the Leu^119^-site is determined in C_12_E_8_ and different monovalent cations (K^+^, Na^+^, and Li^+^) at 0.1 M. The rate increases with increasing Mg^2+^ concentration – binding to a low affinity site favors exposure. The cleavage is faster in Na^+^ and K^+^ as compared with that in Li^+^ or in the absence of monovalent cation, thus K^+^ or Na^+^ binding at the K^+^ site on the P domain^20^,^21^ increases prtK attack at Leu^119^.

*E*2·BeF_3_^−^ cleavage in C_12_E_8_ is inhibited by thapsigargin (TG), which binds tightly to a cavity surrounded by M3, M5, and M7, fixing the arrangement of transmembrane helices with a tightly closed luminal gate^22^,^23^ (Fig. 3a “C_12_E_8_+ TG” for *E*2·BeF_3_^−^). On the other hand, in the BeF_3_^−^-free state with bound TG (“E2·TG”) with Mg^2+^ as well as without Mg^2+^, the 110-kDa ATPase chain is very rapidly cleaved producing p95 and p81/p83 fragments by cleavages at Leu^119^ and at Thr^242^ (p83) and Ala^746^ (p81), respectively, in agreement with previous findings^16^.

### Tryptic T2 (Arg^198^)-site in *E*2·BeF_3_^−^ is completely resistant in C_12_E_8_

The association of the Val^200^ loop (Lys^189^-Lys^205^) on the A domain with the P domain by ionic interactions is crucial for *E*2P structure formation and occurs as a consequence of the A domain’s large rotation during the *E*1PCa_2_ → *E*2P isomeric transition^6^,^7^,^24^. With the changes, the Arg^198^-tryptic T2 site in this loop becomes completely resistant to tryptic attack^4^,^6^. In Fig. 3a, the trypsin proteolysis was performed as described above with prtK. In the BeF_3_^−^-free state with bound TG as a control (“E2·TG”) in which the A and P domains are not fixed, the Arg^198^-site is cleaved producing the A1 and A2 fragments (the A2 fragment is not seen because it is at the gel front) as found previously^6^. In *E*2·BeF_3_^−^, the A1 and A2 fragments are not produced regardless of the presence of C_12_E_8_ and 30 mM Mg^2+^, thus the Arg^198^-site is completely resistant, consistent with association of the A and P domains by an ionic network as seen in the *E*2·BeF_3_^−^ crystal structures^8^,^11^.

### *E*2·BeF_3_^−^ in C_12_E_8_ is completely resistant to prtK at pH 6.0

In Fig. 3e, prtK-proteolysis was performed at pH 6.0 otherwise as in Fig. 3a. At this pH the luminal transport sites are expected to be protonated. No cleavage of the 110 kDa-ATPase chain occurred even in C_12_E_8_ and 30 mM Mg^2+^. The tryptic Arg^198^-site was also completely resistant at pH 6.0 as at pH 7.4 without and with C_12_E_8_ and 30 mM Mg^2+^.

### *E*2·AlF_4_^−^ and *E*2·MgF_4_^2−^ are completely resistant to prtK even in C_12_E_8_ at pH 7.4 and 6.0

*E*2·AlF_4_^−^, the analog for the transition state *E*2~P^‡^ is completely resistant to prtK at pH 7.4 and 6.0 even in the presence of C_12_E_8_ both without and with 30 mM Mg^2+^ (Fig. 3b,f). The Arg^198^-site is also protected from trypsin in all these conditions. *E*2·MgF_4_^2−^, the analog for the product complex (*E*2·P_i_) is completely resistant to prtK and to trypsin in all these conditions as *E*2·AlF_4_^−^ (Fig. 3c,f).

### Hydrophobic nature of the nucleotide/catalytic site revealed by TNP-AMP superfluorescence

TNP-AMP binds to the ATP binding site with a very high affinity and develops an extremely high “superfluorescence” in the *E*2P ground state and its analog *E*2·BeF_3_^− ^4,25. The TNP moiety binds at the adenine position in the N domain and the superfluorescence can be ascribed to a favorable TNP moiety Phe^487^ interaction and site-occlusion that excludes non-specific water and increases hydrophobicity by the contribution of Arg^174^ on the A domain at the A-N interface on the TNP binding pocket^26^. The superfluorescence is completely lost during *E*2P + H_2_O → *E*2~P^‡^, as demonstrated with the change *E*2·BeF_3_^−^ → *E*2·AlF_4_^−^ 4, probably through TNP-Phe^487^ mal-alignment and water influx here. In Fig. 4, the superfluorescence development in *E*2·BeF_3_^−^ upon the TNP-AMP binding at saturating 4 *μ*M was examined without and with C_12_E_8_ at pH 7.4 and 6.0 and various concentrations of Mg^2+^ in 0.1 M K^+^ or Li^+^. There was almost no effect of C_12_E_8_ on superfluorescence development. Specific K^+^ binding on the P domain^20^,^21^ also had virtually no effect (compare the data in K^+^ with those in Li^+^). Increasing Mg^2+^ concentration up to 60 mM caused only slight decrease. The results show that the catalytic/nucleotide site, starting from the *E*2P ground state, is not affected by C_12_E_8_, Mg^2+^, K^+^, and protonation of transport sites.

### *E*2·BeF_3_^−^ in C_12_E_8_ and Mg^2 + ^is resistant to luminal Ca^2+^-induced reverse conversion to *E*1Ca_2_·BeF_3_^−^

The *E*2P ground state possesses luminally partially open low affinity transport sites and luminal Ca^2+^ at sub-mM to ~mM concentration is able to bind and cause reverse isomerization *E*2P + 2Ca^2+^ → *E*2PCa_2_ → *E*1PCa_2_, which contributes to the proper setting of luminal Ca^2+^ concentration through “back-door inhibition”. This reverse process as well as the forward *E*P isomerization is mimicked and characterized with the structural analogs *E*2·BeF_3_^−^ (*E*2P), *E*2·BeF_3_^−^·Ca_2_ (*E*2PCa_2_, the transient intermediate state before the Ca^2+^-release), and *E*1Ca_2_·BeF_3_^−^ (*E*1PCa_2_)^4^,^17^,^18^,^19^. In Figs 5 and 6, the effect of luminal Ca^2+^ on *E*2·BeF_3_^−^ was examined at pH 7.4 in C_12_E_8_ or A23187, various concentrations of Mg^2+^, and 0.1 M K^+^ or Li^+^. Here it should be noted that the *E*1Ca_2_·BeF_3_^−^ complex is not stable and rapidly decomposes to *E*1Ca_2_ in the presence of a high concentration of Ca^2+^ (due to Ca^2+^-substitution at the unoccluded catalytic Mg^2+^ site in *E*1Ca_2_·BeF_3_^− ^17), on the other hand, it is very rapidly isomerized to *E*2·BeF_3_^−^ releasing Ca^2+^ upon the removal or reduction of luminal free Ca^2+^ concentration (to below ~100 *μ*M) as the process mimics the isomeric transition *E*1PCa_2_ → *E*2P + 2Ca^2+ ^17. Also, the *E*1Ca_2_·BeF_3_^−^ complex decomposes to *E*1Ca_2_ upon ADP binding, mimicking the ADP-induced reverse dephosphorylation of *E*1PCa_2_, and upon TNP-AMP binding probably analogous to the ADP-induced process, in contrast to a stable *E*2·BeF_3_^−^ with bound ADP or TNP-AMP^17^.

In Fig. 5, taking these known properties into account, we first determined the overall time course of the Ca^2+^-induced *E*2·BeF_3_^−^ reverse conversion and decomposition to *E*1Ca_2_ (*E*2·BeF_3_^−^ + 2Ca^2+^ → *E*2·BeF_3_^−^·Ca_2_ → *E*1Ca_2_·BeF_3_^−^ → *E*1Ca_2_) by adding an excess EGTA after various times of incubation with 0.5 mM Ca^2+^ thereby converting the remaining *E*1Ca_2_·BeF_3_^−^ to the stable *E*2·BeF_3_^−^ species, and in addition adding TNP-AMP to determine superfluorescence development to estimate the total amount of *E*2·BeF_3_^−^ and *E*1Ca_2_·BeF_3_^−^ species remaining at the time of EGTA addition. In Fig. 6, prtK proteolysis was performed for a short period during the 0.5 mM Ca^2+^ incubation and without the EGTA addition to identify the structural states of *E*P species under representative conditions in Fig. 5 (although the Ca^2+^-induced process proceeds).

First in Fig. 5 where TNP-AMP superfluorescence is examined, we found both with K^+^ and without K^+^ (with LiCl) that the Ca^2+^-induced reverse conversion/decomposition of *E*2·BeF_3_^−^ is considerably slower in C_12_E_8_ than in A23187, and increasing Mg^2+^ to ~20 mM in C_12_E_8_ causes a marked retardation or almost complete inhibition. The retardation by Mg^2+^ in C_12_E_8_ is much stronger and occurs at much lower Mg^2+^ concentration than in A23187. In the absence of both C_12_E_8_ and A23187, *i.e.* with an impermeable SR membrane, no conversion nor decomposition of *E*2·BeF_3_^−^ occurs with Ca^2+^, therefore the Ca^2+^-induced decomposition is due to the Ca^2+^ access from the luminal side as found previously^4^,^17^. Regarding the K^+^ effect, the luminal Ca^2+^-induced conversion/decomposition of *E*2·BeF_3_^−^ is considerably faster in K^+^ than in its absence, therefore specific K^+^ binding^20^,^21^ accelerates the process.

Then in Fig. 6a, prtK-proteolysis was performed to identify the structural state stabilized in C_12_E_8_ with, most typically, 30 mM Mg^2+^ in the absence of K^+^ during luminal Ca^2+^-induced *E*2·BeF_3_^−^ reverse conversion and decomposition. Here, the sample was incubated first with 0.5 mM Ca^2+^ for 10 s, and then with a high concentration of prtK for various times without removal of Ca^2+^. The proteolytic pattern was compared with those of BeF_3_^−^-free *E*1Ca_2_ and of *E*1Ca_2_·BeF_3_^−^ that is formed and stabilized perfectly under the previously identified most appropriate conditions, *i.e.* at pH 7.0 with 0.7 mM Ca^2+^ and 15 mM Mg^2+^ in 0.1 M K^+^ in the absence or presence of A23187 17; in these states, p81/p83 fragments are produced due to cleavage at Thr^242^ (p83) and Ala^746^ (p81) without production of the p95-fragment (Fig. 6b). In C_12_E_8_ and Ca^2+^ (Fig. 6a), *E*2·BeF_3_^−^ both without and with 30 mM Mg^2+^ is degraded slowly as compared with *E*1Ca_2_, producing the stable p95 fragment as seen with *E*2·BeF_3_^−^ in C_12_E_8_ without Ca^2+^ (*cf.* Fig. 3) and a small amount of p81/p83 fragments, which degrade rapidly as the BeF_3_^−^-free *E*1Ca_2_ state. Note also that the 110-kDa ATPase chain degradation is much slower and formation of the rapidly degrading p81/p83 fragments is much less in 30 mM Mg^2+^ than without Mg^2+^. The results show that *E*2·BeF_3_^−^ in C_12_E_8_ and Ca^2+^ is resistant to the luminal Ca^2+^-induced reverse conversion to *E*1Ca_2_·BeF_3_^−^, which can be interpreted as very slow Ca^2+^ binding to luminal transport sites and what slow conversion occurs is markedly retarded by 30 mM Mg^2+^. These results accord with those using superfluorescence as the indicator in Fig. 5.

In the presence of A23187, as seen in Fig. 6a, formation of the p81/p83 fragments from *E*2·BeF_3_^−^ in Ca^2+^ occurs without any p95 fragment, as with *E*1Ca_2_ and *E*1Ca_2_·BeF_3_^−^ in A23187 (*cf.* Fig. 6b) indicating a fast conversion of *E*2·BeF_3_^−^ to *E*1Ca_2_·BeF_3_^−^ without the detergent and with the ionophore. These results together with the retardation by Mg^2+^ of loss of TNP-AMP superfluorescence (Fig. 5) indicate that *E*1Ca_2_·BeF_3_^−^ is formed from *E*2·BeF_3_^−^ without detergent on luminal Ca^2+^ binding and further decomposed to *E*1Ca_2_, and that Mg^2+^ at a high concentration retards the decomposition of *E*1Ca_2_·BeF_3_^−^ to *E*1Ca_2_ probably by inhibiting the Ca^2+^-replacement of Mg^2+^ at the unoccluded catalytic subsite^17^.

### Forward conversion of *E*1Ca_2_·BeF_3_^−^ to *E*2·BeF_3_^−^ is favored in C_12_E_8_

Also in Fig. 6b, it can be seen that under conditions where *E*1Ca_2_·BeF_3_^−^ is perfectly stable in A23187^17^, the addition of C_12_E_8_ in place of A23187 produces the same proteolytic pattern as developed with *E*2·BeF_3_^−^ in C_12_E_8_ and Ca^2+^. The results reveal that the *E*2·BeF_3_^−^ state is produced and stabilized in C_12_E_8_ even under conditions that perfectly stabilize *E*1Ca_2_·BeF_3_^−^ in the absence of C_12_E_8_. This was further verified by superfluorescence development and loss upon TNP-AMP addition in Fig. 6c, which was performed on the basis of previous findings^17^ that *E*1Ca_2_·BeF_3_^−^ rapidly decomposes to the non-fluorescent *E*1Ca_2_ state upon TNP-AMP binding whereas *E*2·BeF_3_^−^ with bound TNP-AMP is stable, and also that the superfluorescence intensity is greater in *E*2·BeF_3_^−^ than in *E*1Ca_2_·BeF_3_^−^ (by approximately 25%). In Fig. 6c, *E*1Ca_2_·BeF_3_^−^ was first formed under the conditions in Fig. 6b without A23187 and C_12_E_8_, and then A23187 or C_12_E_8_ added. After 10 s, superfluorescence upon TNP-AMP addition was recorded. In A23187 or in its absence, superfluorescence development is followed by its rapid loss, which is due to *E*1Ca_2_·BeF_3_^−^ decomposition to *E*1Ca_2_ on TNP-AMP binding^17^. In C_12_E_8_, greater superfluorescence develops and its loss is considerably slower than in A23187. The results show again that in C_12_E_8_, *E*2·BeF_3_^−^ is formed even under conditions that perfectly stabilize *E*1Ca_2_·BeF_3_^−^ (although *E*2·BeF_3_^−^ is slowly decomposed to the non-fluorescent *E*1Ca_2_ state *via E*1Ca_2_·BeF_3_^−^ in high Ca^2+^ and decomposition by TNP-AMP).

### *E*2P hydrolysis

In Fig. 7, the effects of C_12_E_8_, K^+^, and Mg^2+^ on the forward *E*2P hydrolysis rate were examined at pH 7.4 and 6.0. Here *E*2P was first formed in the reverse reaction of hydrolysis from the Ca^2+^-deprived *E*2 state and ^32^P_i_ in 7 mM Mg^2+^ without or with C_12_E_8_ (or with A23187) in 20% (v/v) Me_2_SO, conditions that favor *E*2P formation. Then hydrolysis was initiated by a 20-fold dilution in non-radioactive P_i_, various concentrations of Mg^2+^, and 0.1 M K^+^ (Fig. 7a) or Li^+^ (Fig. 7b) at the desired pH. In K^+^ at pH 7.4, C_12_E_8_ markedly retards hydrolysis as found previously at pH 7.5^27^, and increasing Mg^2+^ concentration in C_12_E_8_ hardly affects the rate (perhaps a slight increase), but the cation decreases the rate in the absence of C_12_E_8_. Because this decrease is observed both without and with A23187 (an ionophore for Ca^2+^ and Mg^2+^) and because Me_2_SO (used for the P_i_-induced *E*2P formation) does not permeabilize the SR membrane, the hydrolysis reaction rate itself is likely affected by Mg^2+^ at the cytoplasmic side. At pH 6.0 in K^+^, hydrolysis is much slower than at pH 7.4, as is well known^28^, and C_12_E_8_ and Mg^2+^ have almost no effect on the slowed rate.

In the absence of K^+^ (Fig. 7b), *E*2P hydrolysis at both pH 7.4 and 6.0 is much slower than in 0.1 M K^+^ (by ~10-fold at the respective pH), in agreement with the well-known acceleration of hydrolysis by specific K^+^ binding on the P domain^20^,^21^. In the absence of K^+^, hydrolysis in C_12_E_8_ is only slightly slower than that without C_12_E_8_. Mg^2+^ at ~10 mM somewhat increases the rate although the rate is still much slower than that in the presence of K^+^. In summary, induction of the detergent-stabilized state strongly inhibits hydrolysis at pH 7.4, but not following protonation of the transport sites at pH 6.0, and only in the presence of K^+^.

## Discussion

**Figure 1:**
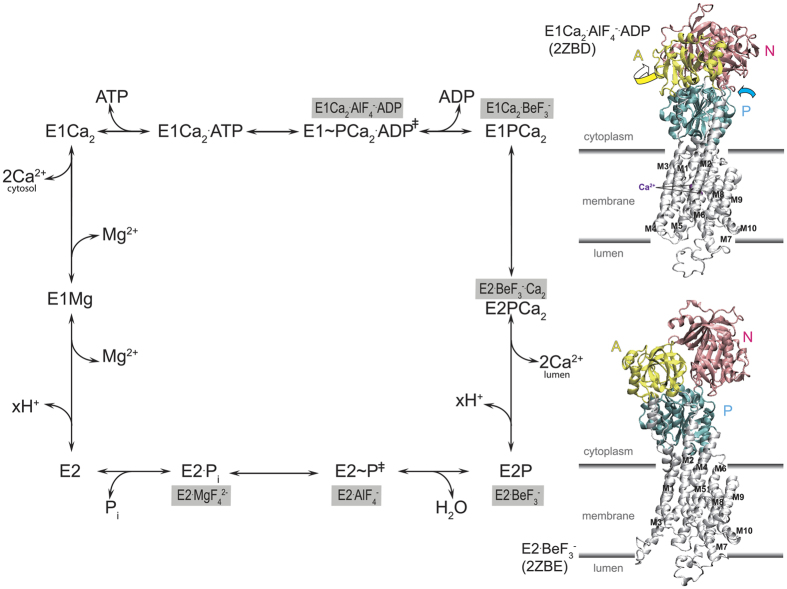
Reaction sequence of Ca^2+^-ATPase. The sequence is shown with intermediates and transition states (*E*1~PCa_2_ADP^‡^ and *E*2~P^‡^). Stable structural analog for each state developed with phosphate analogs BeF_3_^−^, AlF_4_^−^ and MgF_4_^2−^ 4,6,17,19 is shown with gray-highlight. In the crystal structures *E*1Ca_2_·AlF_4_^−^·ADP and *E*2·BeF_3_^−^ (PDB code: 2ZBD^8^ and 2ZBE^8^, respectively), the cytoplasmic domains A (yellow), P (cyan), and N (pink), M1~M10, occluded two Ca^2+^, and membrane position are indicated. Arrows on the domains in *E*1Ca_2_·AlF_4_^−^·ADP indicate their approximate motions to the *E*2·BeF_3_^−^ structure to show changes in *E*1PCa_2_ → *E*2P+ 2Ca^2+^ as an available model.

**Figure 2:**
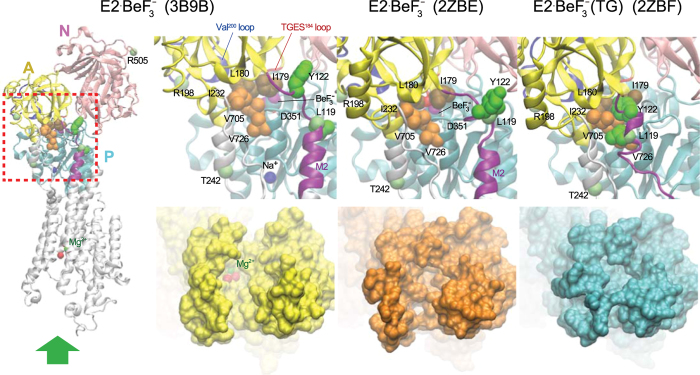
Crystal structures *E*2·BeF_3_^−^ and *E*2·BeF_3_^−^(TG). Structures *E*2·BeF_3_^−^ with bound Mg^2+^ at the transport sites (formed at pH 7.0 and 50 mM Mg^2+^), *E*2·BeF_3_^−^ with most probably protonated transport sites (formed at pH 5.7), and *E*2·BeF_3_^−^(TG) (PDB code: 3B9B^11^, 2ZBE^8^, 2ZBF^8^, respectively) are shown as a cartoon model. The cytoplasmic region indicated by the red broken line on the whole molecule of *E*2·BeF_3_^−^ with bound Mg^2+^ is enlarged in the three top panels. In the three bottom panels, the view of transport sites from the luminal side as indicated by a large green arrow is shown. The A, P, and N domains and cytoplasmic part of M2 are yellow, cyan, pink, and purple, respectively. The Mg^2+^ and water molecules at the Ca^2+^ binding sites (transport sites) and Na^+^ bound at the K^+^ (Na^+^) site on the P domain are green, red, and blue spheres, respectively. The seven residues involved in the formation of Tyr^122^-hydrophobic cluster, Y122-HC (Leu^119^/Tyr^122^ on M2, Ile^179^/Leu^180^ on the A domain, Val^705^/Val^726^ on the P domain, and Ile^232^ on the A/M3-linker) are shown with van der Waals spheres, and colored green (Leu^119^/Tyr^122^), brown (Ile^179^/Leu^180^), and orange (Val^705^/Val^726^/Ile^232^). The BeF_3_^−^ coordinated in the catalytic site behind the residues involved in Y122-HC is shown by a space-filling model (cyan for beryllium and purple for fluoride) and Asp^351^ (the auto-phosphorylation site) is shown in a ball-stick model in the panels (note that they are obscured by Y122-HC in *E*2·BeF_3_^−^(TG)). The Mg^2+^ bound to the catalytic site is not depicted as it is also hidden by Y122-HC. The TGES^184^ loop and Val^200^ loop (Lys^189^-Lys^205^) are colored by a red loop and a blue loop, respectively in all panels. The prtK-cleavage sites at Leu^119^ and Thr^242^ and the trypsin-cleavage sites at Arg^198^ and Arg^505^ are indicated (backbone carbon).

Ca^2+^ transport by Ca^2+^-ATPase includes phosphorylated intermediates where Ca^2+^ is occluded at the transport sites and then released to the lumen, *i.e. E*1P[Ca_2_] → *E*2P + Ca^2+^. During this process the A domain swings around and engages with the P domain and neck region of the protein at the cytoplasmic part of M2 (Fig. 1). The A-domain rotation inclines the P-domain by pulling an A/M1′-link, pushing M4 down towards the lumen to release the Ca^2+^ 8,18,19. There is evidence that the gathering and interaction of A and P domains at the cytoplasmic part of M2 occurs progressively. Namely, changes, which are linked to deocclusion and opening of the luminal access channel with an affinity reduction, are followed by constrictions to limit access, protonation, and finally closure, and all these changes are synchronized with catalytic site preparations for hydrolysis^4^,^13^,^15^,^17^,^18^,^19^. Part of the development is seen with the Leu^119^ prtK cleavage site, being exposed in *E*2PCa_2_, hidden in *E*2P, *E*2~P^‡^ and *E*2·P_i_, and exposed again in *E*2^4^,^18^,^19^. We found here that non-solubilizing concentrations of C_12_E_8_ uncovers the Leu^119^ prtK site of *E*2P, as depicted in its analog *E*2·BeF_3_^−^. This indicates that membrane perturbation drives the intermediate towards one more like that with bound Ca^2+^, and points to an earlier catalytic intermediate with a looser arrangement in the head region, as expected for early engagement of the rotated A domain. The responsiveness of *E*2P to membrane perturbation and the detergent-induced state to ligand binding (Ca^2+^, Mg^2+^, K^+^, H^+^, and TG) through changes in exposure of the Leu^119^ prtK site at the cytoplasmic part of M2 points to flexible and rather unstable forms. These properties are due most probably not only to its unoccupied transport sites and associated circle of negative charges, but also to a loose meeting of domains and neck region with largely unsecured interactions at the cytoplasmic part of M2. The downward thrust of M4 (by a full turn of an α-helix^8^), together with M3, is probably partly stabilized by surrounding phospholipids and insertion of non-ionic detergent between them could be disruptive. In the head region the interactions at Leu^119^ involve the formation of Y122-HC, a hydrophobic interaction network of Tyr^122^/Leu^119^ with the A and P domains and A/M3-linker involving seven residues (Fig. 2). As mentioned above, the interactions are likely progressive, loose at first as the A domain engages followed by incremental tightening in *E*2P to the fully stabilized state in *E*2~P^‡^ and *E*2·P_i_. Indeed, in the *E*2·BeF_3_^−^ crystal structures (formed in the presence of C_12_E_8_) with the bound Mg^2+^ or with protonation without the Mg^2+^, Leu^119^/Tyr^122^ on M2 are close but not yet associated with the five other gathered residues involved in Y122-HC formation. The knitting of Leu^119^ and Tyr^122^ with the other residues is seen in the crystal structures of analogs of the next intermediates, *E*2~P^‡^ and *E*2·P_i_. Accumulating interactions fit perfectly with the staggered changes at the luminal transport sites, from closed to open to closed again.

**Figure 3:**
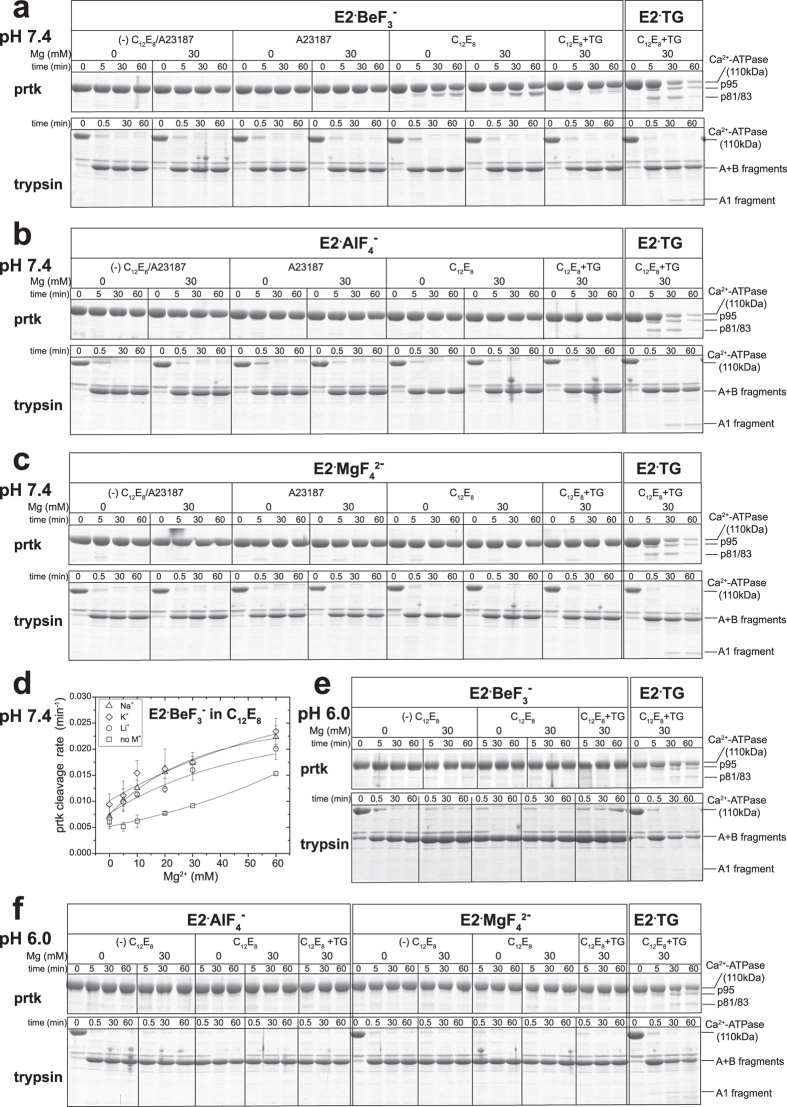
Effects of C_12_E_8_ and various factors on proteolysis of *E*2·BeF_3_^−^, *E*2·AlF_4_^−^, and *E*2·MgF_4_^2−^. The proteolysis was performed for various times with prtK and trypsin as indicated with *E*2·BeF_3_^−^ (**a,e**), *E*2·AlF_4_^−^ (**b,f**), and *E*2·MgF_4_^2−^ (**c,f**) of SR vesicles in the presence or absence of 0.15 mg/ml C_12_E_8_ or 15 *μ*M A23187 in 50 mM MOPS/Tris pH 7.4 (**a–c**) or MES/Tris pH 6.0 (**e,f**), 0.1 M KCl, 1 mM EGTA, and 0 or 30 mM MgCl_2_ without or with 4 *μ*M TG (“TG”), as indicated. The “E2·TG” state of SR vesicles un-treated with the metal fluoride was subjected to the proteolysis as a control. In (**d**), the rate of prtK digestion of 110 kDa-ATPase chain in C_12_E_8_ at pH 7.4 was determined at various concentrations of MgCl_2_ in 0.1 M KCl, NaCl, or LiCl or in the absence of these salts, otherwise as in (**a**) and as described under “METHODS”. The fragments indicated on the right of a panel are p95 produced by the prtK-cleavage at the Leu^119^-site on M2, p81/p83 produced by the prtK-cleavage at the Thr^242^-site on A/M3-linker (p83) and Ala^746^ on M5 (p81)^16^,^39^, and the tryptic A1 fragment produced by cleavage at the Arg^198^-site on the A fragment (N-terminal half), which is formed very rapidly together with the B fragment (C-terminal half) by cleavage at Arg^505^-site^40^.

**Figure 4:**
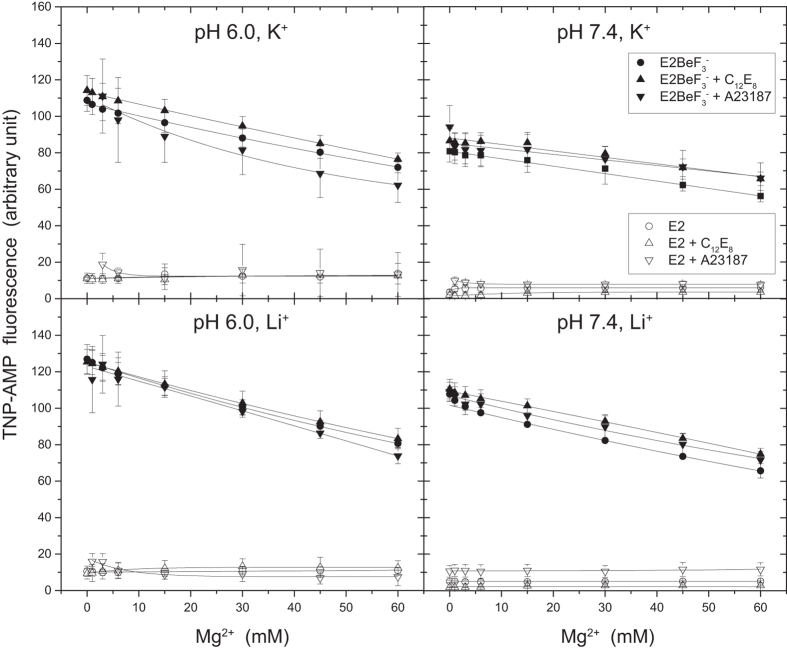
Hydrophobic property at nucleotide/catalytic site in *E*2·BeF_3_^−^ revealed by TNP-AMP superfluorescence. *E*2·BeF_3_^−^ or the BeF_3_^−^-free Ca^2+^-ATPase (*E2*) in SR vesicles (0.06 mg protein/ml) were incubated at 25 °C for 3 min in 0.5 mM EGTA, 30 mM MES/Tris (pH 6.0) or MOPS/Tris (pH 7.4), 0.1 M KCl or LiCl, and 0–60 mM MgCl_2_ with or without 0.02 mg/ml C_12_E_8_ and/or 2.5 *μ*M A23187, as indicated in the figure. Subsequently, TNP-AMP at saturating 4 *μ*M was added. The fluorescence intensity was obtained by subtracting the protein background level without TNP-AMP and the level of 4 *μ*M TNP-AMP without SR vesicles, and plotted *versus* Mg^2+^ concentration.

**Figure 5:**
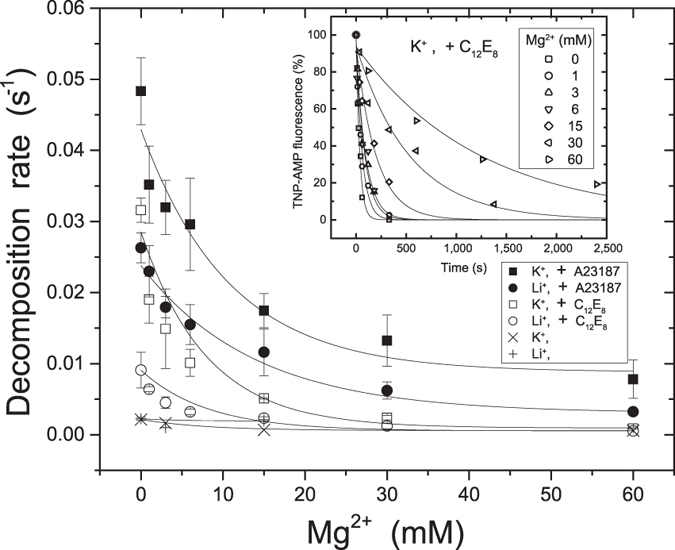
Luminal Ca^2+^-induced reverse conversion and decomposition of *E*2·BeF_3_^−^ determined by loss of TNP-AMP superfluorescence. *E*2·BeF_3_^−^ in SR vesicles was incubated at 25 °C for 3 min in 30 mM MOPS/Tris (pH 7.4), 0.1 M KCl or LiCl, 0.5 mM EGTA, 0–60 mM MgCl_2_ with or without 0.15 mg/ml C_12_E_8_ or 15 *μ*M A23187, as indicated. Subsequently Ca^2+^ was added to give 0.5 mM free concentration and incubated for various times, then diluted 10-fold with the above solution containing 5 mM EGTA without Ca^2+^. At 30 s after dilution, 4 *μ*M TNP-AMP was added to determine the superfluorescence intensity. The representative time courses of loss of superfluorescence in C_12_E_8_ in 0.1 M K^+^ are shown in *inset*. The rate of Ca^2+^-induced *E*2·BeF_3_^−^ decomposition was determined by least-squares fit of a single exponential to the time course and plotted *versus* the Mg^2+^ concentration.

**Figure 6:**
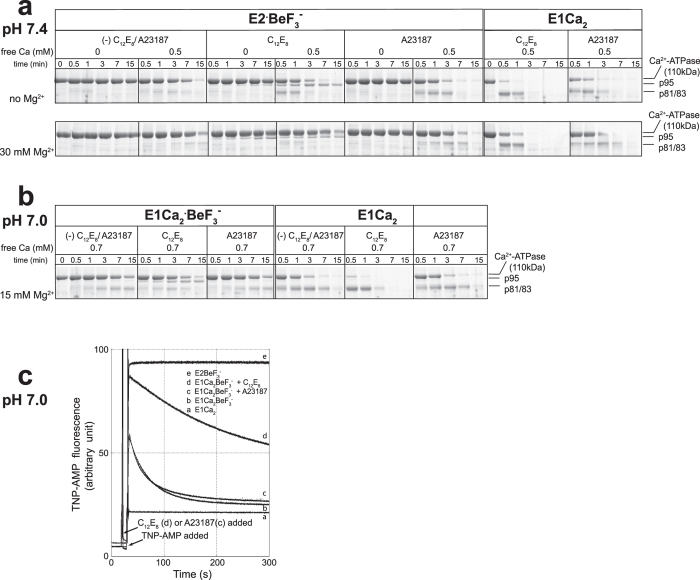
Luminal Ca^2+^-effect on *E*2·BeF_3_^−^ in C_12_E_8_ (a) and formation and stabilization of *E*2·BeF_3_^−^ in forward conversion from *E*1Ca_2_·BeF_3_^−^ in C_12_E_8_ (b,c). (**a**) *E*2·BeF_3_^−^ in SR vesicles was incubated without or with 0.15 mg/ml C_12_E_8_ or with 15 *μ*M A23187 at 25 °C for 3 min in 30 mM MOPS/Tris (pH 7.4), 0.1 M LiCl, 0.5 mM EGTA, and 0 (upper panel) or 30 mM MgCl_2_ (lower panel), then Ca^2+^ was added to give 0.5 mM free concentration. After 10 s, prtK was added at 0.5 mg/ml and incubated for indicated times. As a control, the BeF_3_^−^-free Ca^2+^-ATPase in SR vesicles (“E1Ca_2_”) was subjected to the proteolysis in 0.5 mM free Ca^2+^. (**b**) The prtK proteolysis was performed under the conditions that produce and perfectly stabilize *E*1Ca_2_·BeF_3_^−^ 17, *i.e.* 30 mM MOPS/Tris (pH 7.0), 0.1 M KCl, 15 mM MgCl_2_, and 0.7 mM CaCl_2_ in the presence of 100 *μ*M BeCl_2_ and 2 mM KF without and with 15 *μ*M A23187, and the effect of C_12_E_8_ was examined by including C_12_E_8_ without A23187, otherwise as in (**a**). The BeF_3_^−^-free Ca^2+^-ATPase (“E1Ca_2_”) in A23187 and in C_12_E_8_ was subjected to proteolysis otherwise as above. Note that the slow decomposition of *E*2·BeF_3_^−^ in Ca^2+^ in the absence of A23187 and C_12_E_8_ (**a**) is probably due to slow Ca^2+^ permeation into the SR vesicles lumen^17^. (**c**) *E*1Ca_2_·BeF_3_^−^ was produced by incubating SR vesicles for 30 min with 100 *μ*M BeCl_2_ and 2 mM KF in the absence of A23187 and C_12_E_8_ otherwise as in (**b**), then C_12_E_8_ or A23187 was added to give 0.02 mg/ml and 2.5 *μ*M, respectively. At 10 s after this addition, TNP-AMP was added to give a saturating 4 *μ*M, and the fluorescence monitored; trace b, without C_12_E_8_ and A23187; traces c and d, in A23178 and in C_12_E_8_, respectively. Trace e, the fluorescence monitored with *E*2·BeF_3_^−^ in the presence of 2 mM EGTA without adding Ca^2+^. Trace a, the non-superfluorescent *E*1Ca_2_ level (BeF_3_^−^-free Ca^2+^-ATPase) in 4 *μ*M TNP-AMP.

Stabilization of the early detergent-induced state is seen in the forward direction of catalysis coming from *E*1PCa_2_ (*E*1Ca_2_·BeF_3_^−^) and in the backward direction with Ca^2+^ binding to the luminal sites of *E*2P (*E*2·BeF_3_^−^), using both TNP-AMP superfluorescence and the prtK sites as probes. Our results suggest that the *E*2·BeF_3_^−^ structural state favored in C_12_E_8_ and stabilized by Mg^2+^ represents one between *E*1PCa_2_ and Ca^2+^-released *E*2P, *i.e.* the transient *E*2P state immediately following Ca^2+^ release denoted as *E*2P^∗^ with luminally open and vacant low affinity transport sites (*E*2P^∗^Ca_2_ → *E*2P^∗^ in Fig. 8). C_12_E_8_ stabilizes the *E*2P^∗^ state and thereby retards both the luminal Ca^2+^-induced reverse conversion and the forward hydrolysis of *E*2P at pH 7.4. Mg^2+^ binding probably prevents luminal Ca^2+^-access and consequent reverse conversion (Figs 3,5 and 6). This Mg^2+^ is likely at or near the luminally open Ca^2+^ transport sites (in addition to Mg^2+^ occluded at the catalytic subsite in *E*2·BeF_3_^−^ and *E*2P) as actually seen in the *E*2·BeF_3_^−^ crystal produced in a high concentration of Mg^2+ ^11. The Mg^2+^ probably manifests itself in the competitive inhibition by Mg^2+^ of luminal Ca^2+^-induced reverse isomerization *E*2P+ 2Ca^2+^ → *E*1PCa_2_^29^. Notably also, the dephosphorylated *E*1 state is able to accommodate one Mg^2+^ at the transport sites and forms *E*1·Mg, which favors high affinity Ca^2+^-binding resulting in a rapid *E*2 → *E*1·Mg → *E*1Ca_2_ transition^30^,^31^ (Fig. 1). Thus it seems that Mg^2+^ binds at the empty transport sites both in the unphosphorylated and phosphorylated states and modifies transport function.

The *E*2·BeF_3_^−^ structures revealed in C_12_E_8_ and in A23187 at pH 7.4 reflect *E*2P^∗^ and *E*2P respectively in Fig. 8 on the basis of prtK-resistance. Analysis of the Mg^2+^ inhibition of luminal Ca^2+^-induced reverse conversion of *E*2·BeF_3_^−^ in Figs 5 and 6 indicates that Mg^2+^ accesses *E*2P^∗^ with a much higher affinity than *E*2P. Thus the transport sites appear more open and accessible to Mg^2+^ on the luminal side in the Leu^119^-site cleavable *E*2P^∗^ state than in the prtK-resistant *E*2P ground state. In fact, in the *E*2·BeF_3_^−^ crystal with bound Mg^2+^ at the transport sites, the sites are actually more open to the lumen than in the structure without Mg^2+^ (Fig. 2). Note also that in *E*2·AlF_4_^−^ and *E*2·MgF_4_^2−^ (*E*2~P^‡^ and *E*2·P_i_) and in *E*2·BeF_3_^−^ with bound TG, Ca^2+^ cannot bind as the luminal gate is tightly closed^4^,^7^,^8^, and the Leu^119^-site is completely resistant to prtK regardless of the presence of C_12_E_8_ (Fig. 3). These findings suggest that the structural change reflected by prtK resistance at Leu^119^ is associated with luminal gating, supporting the above conclusion that substantial luminal gate closure occurs in *E*2P^∗^ → *E*2P, which probably involves gathering of Leu^119^/Tyr^122^ with the engaged A and P domains to accomplish the Y122-HC network. Then the passage is completely sealed in *E*2~P^‡^ and *E*2·P_i_ (*E*2·AlF_4_^−^ and *E*2·MgF_4_^2−^)^4^.

**Figure 7:**
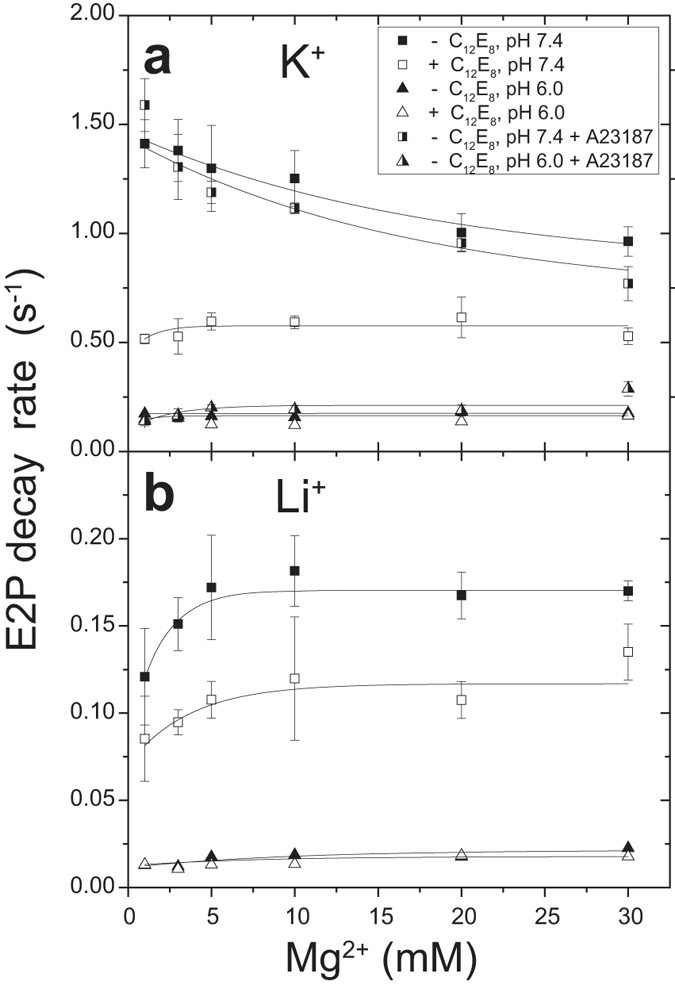
Effects of C_12_E_8_ and various factors on *E*2P hydrolysis. SR vesicles were phosphorylated with 0.1 mM ^32^P_i_ at 25 °C for 10 min in 5 *μ*l of a mixture containing 0.3 mg protein/ml with or without 3 *μ*M A23187 as indicated, 1 mM EGTA, 7 mM MgCl_2_, 30 mM MOPS/Tris (pH 7.4) or MES/Tris (pH 6.0), and 20% (v/v) Me_2_SO. The mixture was then cooled, and a small volume of C_12_E_8_ was added to give 0.1 mg/ml (1/3 (w/w) of the protein) to the indicated samples. Subsequently, the samples were diluted at 0 °C by the addition of 95 *μ*l of a mixture containing 0.1 mM non-radioactive P_i_, 105 mM KCl (**a**) or LiCl (**b**), 1 mM EGTA, 1–30 mM MgCl_2_, and 50 mM MOPS/Tris (pH 7.4) or MES/Tris (pH 6.0), as indicated with different symbols. The *E*2P hydrolysis rate was determined as described under “METHODS” and plotted *versus* Mg^2+^concentration. Note the difference in the scale of the ordinate in (**a**) and (**b**).

**Figure 8:**
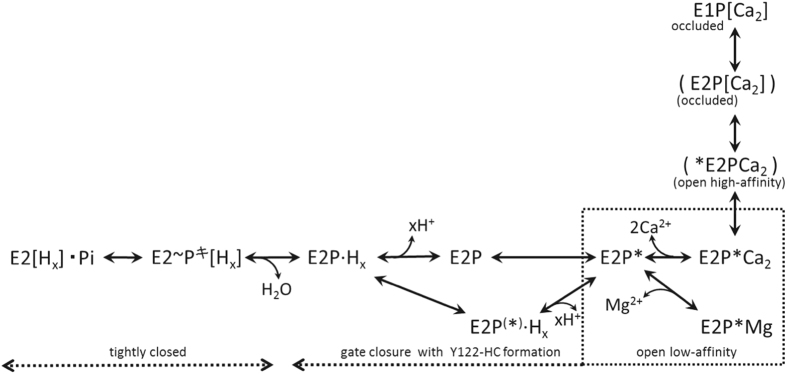
*E*P processing and gating. The effects of C_12_E_8_, luminal Ca^2+^, and Mg^2+^ found in this study are summarized. *E*2P[Ca_2_] (*E*2P with occluded Ca^2+^) and ^∗^*E*2PCa_2_ (*E*2P with luminally open gate and with bound Ca^2+^ yet at a high affinity) were previously identified by the elongation of the A/M1′-linker^18^,^19^ and by substitutional mutation of Leu^119^ and Tyr^122 ^15, but they are transient intermediates and have never been trapped or identified in wild type^15^,^18^,^19^; therefore they are shown in brackets. The *E*2P structural states found in this study at pH 7.4, the Leu^119^-cleavable state and the prtK-resistant state are denoted as *E*2P^∗^ and *E*2P, respectively. The prtK-resistant state found in C_12_E_8_ at pH 6.0 (*i.e.* with protonation of the transport sites) is denoted as *E*2P(^∗^). Note that Y122-HC formation on gathering of Leu^119^/Tyr^122^ on M2 with engaged A and P domains occurs progressively during *E*2P processing and couples with luminal gating (see more in “Discussion”).

Previous kinetic analysis of the luminal Ca^2+^-induced reverse isomerization *E*2P + 2Ca^2+^ → *E*1PCa_2_ indicated^14^ that the luminal Ca^2+^ access to the transport sites in *E*2P is rate-limiting. This is described in Fig. 8 with the equilibrium *E*2P^∗^ ↔ *E*2P, where the former state is more open and the latter relatively closed. This view agrees with our finding on the Ca^2+^ release kinetics *E*1PCa_2_ → *E*2PCa_2_ → *E*2P+ 2Ca^2+^ 15 that the *E*2P structure proceeds from a luminally open state for Ca^2+^ release (corresponding to *E*2P^∗^ in Fig. 8) to a closed state (*E*2P) with the structural contribution of Leu^119^/Tyr^122^. The observation that Mg^2+^ hardly alters the forward *E*2P hydrolysis rate in C_12_E_8_ (Fig. 7a) can be accounted for by a rapid Mg^2+^ binding/release relative to the hydrolysis reaction process, and implies that Mg^2+^ binding favors the forward reaction.

At pH 6.0 in which the transport sites are protonated, the Leu^119^-site is completely resistant to prtK regardless of the presence of C_12_E_8_, and the *E*2P hydrolysis rate is not affected by C_12_E_8_. In Fig. 8, the protonated structural state with the prtK-resistance revealed in C_12_E_8_ is denoted as *E*2P(^∗^) to be discriminated from the prtK-cleavable *E*2P^∗^ state without protonation. Protonation neutralizes charges at the Ca^2+^-binding sites and stabilizes the arrangement of transmembrane helices *via* a hydrogen bonding network^8^, which lowers Ca^2+^-accessibility (without completely closing the gate as seen in the *E*2·BeF_3_^−^ crystal formed at pH 5.7^8^). The protonated state proceeds promptly to subsequent hydrolysis with tight gate closure *E*2P+ H_2_O → *E*2~P^‡^ (*E*2·BeF_3_^−^ → *E*2·AlF_4_^−^), as indicated previously by kinetic analysis of *E*2P hydrolysis^5^.

K^+^ in the presence of C_12_E_8_ accelerates both forward *E*2P hydrolysis and luminal Ca^2+^-induced reverse conversion of *E*2·BeF_3_^−^ (Figs 5 and 7). These findings are in complete agreement with the known role of specific K^+^ binding on the P domain in accelerating both forward hydrolysis^20^,^21^ and luminal Ca^2+^-induced reverse conversion of *E*2P^14^. K^+^ binding likely destabilizes both *E*2P and *E*2P^∗^ in Fig. 8, thus promoting rapid transport.

Finally, induction of the detergent-stabilized state, an early intermediate to ground state *E*2P, shows how phospholipids are intimately involved in the latter’s stabilization. Membrane perturbation effects during the transport cycle may be under-appreciated as fundamental to the mechanism.

## Methods

### Preparation of SR vesicles and treatment with BeF_x_, AlF_x_, and MgF_x_

SR vesicles were prepared from rabbit skeletal muscle as described^32^,^33^, in which all the methods were carried out in accordance with institutional laws and regulations of the Asahikawa Medical University and the experimental protocols were approved by the Animal Experimentation Ethics Committee of the Asahikawa Medical University (license number 16006). The content of the phosphorylation site in the vesicles and the Ca^2+^-dependent ATPase activity were determined as described^32^,^33^. *E*2·BeF_3_^−^, *E*2·AlF_4_^−^, and *E*2·MgF_4_^2−^ were produced by incubating the SR vesicles with the respective metal fluoride and by washing the unbound ligands as described previously^4^.

### Formation and hydrolysis of *E*2P

The SR vesicles were phosphorylated with 0.1 mM ^32^P_i_ at 25 °C for 10 min in 20% (v/v) Me_2_SO in the absence of Ca^2+^, after which the samples were cooled and diluted 20-fold by a solution containing 2.1 mM non-radioactive P_i_ to initiate the hydrolysis of ^32^P_i_-labeled *E*2P, otherwise as described in detail in the legend to Fig. 7. The reaction was quenched with ice-cold trichloroacetic acid containing P_i_. The precipitated proteins were separated by 5% SDS-PAGE at pH 6.0 according to Weber and Osborn^34^. The radioactivity associated with the separated Ca^2+^-ATPase was quantified by digital autoradiography as described^35^. Rapid kinetics measurement of hydrolysis was performed with a handmade rapid mixing apparatus and the rate of hydrolysis was determined with the least-squares fit to a single exponential, as described^35^.

### Proteolytic analysis

SR vesicles (0.45 mg/ml protein) were subjected to proteolysis at 25 °C by addition of trypsin (at 0.3 mg/ml, L-1-tosylamido-2-phenylethyl chloromethyl ketone-treated) or proteinase K (prtK, at 0.1 mg/ml, Sigma) as described previously^6^,^16^, otherwise as indicated in the figure legends. The proteolysis was terminated by trichloroacetic acid, and the samples were subjected to Laemmli SDS-polyacrylamide gel electrophoresis^36^, and densitometric analyses of the gels stained with Coomassie Brilliant Blue R-250, as described^6^,^16^. The degradation rate of 110-kDa ATPase chain with prtK was determined by least-squares fit of a single exponential to the time course (0–150 min) as described previously^16^.

### Fluorescence measurements

The TNP-AMP fluorescence of the Ca^2+^-ATPase (0.06 mg/ml protein, TNP-AMP from Molecular Probes® Life Technologies) was measured on a RF-5300PC spectrofluorophotometer (Shimadzu, Kyoto, Japan) with excitation and emission wavelengths 408 and 540 nm (with band widths 5 and 10 nm), as described previously^4^.

### Miscellaneous

Protein concentrations were determined by the method of Lowry *et al*.^37^ with bovine serum albumin as a standard. Three-dimensional models of the enzyme were reproduced by the program VMD^38^. The values presented are the mean ± s.d. (n = 3–4).

## Additional Information

**How to cite this article**: Danko, S. *et al*. Membrane Perturbation of ADP-insensitive Phosphoenzyme of Ca^2+^-ATPase Modifies Gathering of Transmembrane Helix M2 with Cytoplasmic Domains and Luminal Gating. *Sci. Rep.* **7**, 41172; doi: 10.1038/srep41172 (2017).

**Publisher's note:** Springer Nature remains neutral with regard to jurisdictional claims in published maps and institutional affiliations.

## References

[b1] Toyoshima C. Structural aspects of ion pumping by Ca^2+^-ATPase of sarcoplasmic reticulum. Arch. Biochem. Biophys. 476, 3–11 (2008). 18455499 10.1016/j.abb.2008.04.017

[b2] Toyoshima C. How Ca^2+^-ATPase pumps ions across the sarcoplasmic reticulum membrane. Biochim. Biophys. Acta 1793, 941–946 (2009). 19010358 10.1016/j.bbamcr.2008.10.008

[b3] Møller J. V., Olesen C., Winther A.-M. L. & Nissen P. The sarcoplasmic Ca^2+^-ATPase: design of a perfect chemi-osmotic pump. Q. Rev. Biophys. 43, 501–566 (2010). 20809990 10.1017/S003358351000017X

[b4] Danko S., Yamasaki K., Daiho T. & Suzuki H. Distinct natures of beryllium fluoride-bound, aluminum fluoride-bound, and magnesium fluoride-bound stable analogues of an ADP-insensitive phosphoenzyme intermediate of sarcoplasmic reticulum Ca^2+^-ATPase. J. Biol. Chem. 279, 14991–14998 (2004). 14754887 10.1074/jbc.M313363200

[b5] Seekoe T., Peall S. & McIntosh D. B. Thapsigargin and dimethyl sulfoxide activate medium Pi - HOH oxygen exchange catalyzed by sarcoplasmic reticulum Ca^2+^-ATPase. J. Biol. Chem. 276, 46737–46744 (2001). 11595736 10.1074/jbc.M106320200

[b6] Danko S., Yamasaki K., Daiho T., Suzuki H. & Toyoshima C. Organization of cytoplasmic domains of sarcoplasmic reticulum Ca^2+^-ATPase in E_1_P and E_1_ATP states: a limited proteolysis study. FEBS Lett. 505, 129–135 (2001). 11557055 10.1016/s0014-5793(01)02801-0

[b7] Toyoshima C., Nomura H. & Tsuda T. Lumenal gating mechanism revealed in calcium pump crystal structures with phosphate analogues. Nature 432, 361–368 (2004). 15448704 10.1038/nature02981

[b8] Toyoshima C., Norimatsu Y., Iwasawa S., Tsuda T. & Ogawa H. How processing of aspartylphosphate is coupled to lumenal gating of the ion pathway in the calcium pump. Proc. Natl. Acad. Sci. USA 104, 19831–19836 (2007). 18077416 10.1073/pnas.0709978104 PMC2148383

[b9] Daiho T., Yamasaki K., Danko S. & Suzuki H. Second transmembrane helix (M2) and long range coupling in Ca^2+^-ATPase. J. Biol. Chem. 289, 31241–31252 (2014). 25246522 10.1074/jbc.M114.584086 PMC4223325

[b10] Olesen C., Sørensen T. L., Nielsen R. C., Møller J. V. & Nissen P. Dephosphorylation of the calcium pump coupled to counterion occlusion. Science 306, 2251–2255 (2004). 15618517 10.1126/science.1106289

[b11] Olesen C. . The structural basis of calcium transport by the calcium pump. Nature 450, 1036–1042 (2007). 18075584 10.1038/nature06418

[b12] Yamasaki K., Daiho T., Danko S. & Suzuki H. Multiple and distinct effects of mutations of Tyr^122^, Glu^123^, Arg^324^, and Arg^334^ involved in interactions between the top part of second and fourth transmembrane helices in sarcoplasmic reticulum Ca^2+^-ATPase. J. Biol. Chem. 279, 2202–2210 (2004). 14578351 10.1074/jbc.M309398200

[b13] Wang G., Yamasaki K., Daiho T. & Suzuki H. Critical hydrophobic interactions between phosphorylation and actuator domains of Ca^2+^-ATPase for hydrolysis of phosphorylated intermediate. J. Biol. Chem. 280, 26508–26516 (2005). 15901722 10.1074/jbc.M503789200

[b14] Yamasaki K., Wang G., Daiho T., Danko S. & Suzuki H. Roles of Tyr^122^-hydrophobic cluster and K^+^ binding in Ca^2+^-releasing process of ADP-insensitive phosphoenzyme of sarcoplasmic reticulum Ca^2+^-ATPase. J. Biol. Chem. 283, 29144–29155 (2008). 18728008 10.1074/jbc.M804596200 PMC2662013

[b15] Yamasaki K., Daiho T., Danko S. & Suzuki H. Assembly of a Tyr^122^ hydrophobic cluster in sarcoplasmic reticulum Ca^2+^-ATPase synchronizes Ca^2+^ affinity reduction and release with phosphoenzyme isomerization. J. Biol. Chem. 290, 27858–27879 (2015). 10.1074/jbc.M115.693770 PMC4646029 26442589

[b16] Danko . ADP-insensitive phosphoenzyme intermediate of sarcoplasmic reticulum Ca^2+^-ATPase has a compact conformation resistant to proteinase K, V8 protease and trypsin. FEBS Lett. 489, 277–282 (2001). 11165264 10.1016/s0014-5793(01)02111-1

[b17] Danko S., Daiho T., Yamasaki K., Liu X. & Suzuki H. Formation of the stable structural analog of ADP-sensitive phosphoenzyme of Ca^2+^-ATPase with occluded Ca^2+^ by beryllium fluoride. J. Biol. Chem. 284, 22722–22735 (2009). 19561071 10.1074/jbc.M109.029702 PMC2755681

[b18] Daiho T., Yamasaki K., Danko S. & Suzuki H. Critical role of Glu^40^-Ser^48^ loop linking actuator domain and first transmembrane helix of Ca^2+^-ATPase in Ca^2+^ deocclusion and release from ADP-insensitive phosphoenzyme. J. Biol. Chem. 282, 34429–34447 (2007). 17881350 10.1074/jbc.M707665200

[b19] Daiho T., Danko S., Yamasaki K. & Suzuki H. Stable structural analog of Ca^2+^-ATPase ADP-insensitive phosphoenzyme with occluded Ca^2+^ formed by elongation of A-domain/M1’-linker and beryllium fluoride binding. J. Biol. Chem. 285, 24538–24547 (2010). 20529842 10.1074/jbc.M110.144535 PMC2915690

[b20] Shigekawa M. & Pearl L. J. Activation of calcium transport in skeletal muscle sarcoplasmic reticulum by monovalent cations. J. Biol. Chem. 251, 6947–6952 (1976). 136443

[b21] Sorensen T. L. . Localization of a K^+^ -binding site involved in dephosphorylation of the sarcoplasmic reticulum Ca^2+^-ATPase. J. Biol. Chem. 279, 46355–46358 (2004). 15383548 10.1074/jbc.C400414200

[b22] Inesi G., Lewis D., Toyoshima C., Hirata A. & de Meis L. Conformational fluctuations of the Ca^2+^-ATPase in the native membrane environment. Effects of pH, temperature, catalytic substrates, and thapsigargin. J. Biol. Chem. 283, 1189–1196 (2008). 17993458 10.1074/jbc.M707189200

[b23] Toyoshima C. & Nomura H. Structural changes in the calcium pump accompanying the dissociation of calcium. Nature 418, 605–611 (2002). 12167852 10.1038/nature00944

[b24] Kato S. . Val^200^ residue in Lys^189^–Lys^205^ outermost loop on the A domain of sarcoplasmic reticulum Ca^2+^-ATPase is critical for rapid processing of phosphoenzyme intermediate after loss of ADP sensitivity. J. Biol. Chem. 278, 9624–9629 (2003). 12496291 10.1074/jbc.M208861200

[b25] Dupont Y. & Pougeois R. Evaluation of H_2_O activity in the free or phosphorylated catalytic site of Ca^2+^-ATPase. FEBS Lett. 156, 93–98 (1983). 6221945 10.1016/0014-5793(83)80255-5

[b26] Toyoshima C., Yonekura S., Tsueda J. & Iwasawa S. Trinitrophenyl derivatives bind differently from parent adenine nucleotides to Ca^2+^-ATPase in the absence of Ca^2+^. Proc. Natl. Acad. Sci. USA. 108, 1833–1838 (2011). 21239683 10.1073/pnas.1017659108 PMC3033254

[b27] Champeil P. . Kinetic characterization of the normal and detergent-perturbed reaction cycles of the sarcoplasmic reticulum calcium pump. Rate-limiting step(s) under different conditions. J. Biol. Chem. 261, 16372–16384 (1986). 2946685

[b28] Wakabayashi S., Ogurusu T. & Shigekawa M. Modulation of the hydrolysis rate of the ADP-insensitive phosphoenzyme of the sarcoplasmic reticulum ATPase by H^+^ and Mg^2+^. J. Biol. Chem. 262, 9121–9129 (1987). 2954958

[b29] Bishop J. E. & Al-Shawi M. K. Inhibition of sarcoplasmic reticulum Ca^2+^-ATPase by Mg^2+^ at high pH. J. Biol. Chem. 263, 1886–1892 (1988). 2962998

[b30] Toyoshima C. . Crystal structures of the calcium pump and sarcolipin in the Mg^2+^ -bound E1 state. Nature 495, 260–264 (2013). 23455422 10.1038/nature11899

[b31] Winther A. M. . The sarcolipin-bound calcium pump stabilizes calcium sites exposed to the cytoplasm. Nature 495, 265–269 (2013). 23455424 10.1038/nature11900

[b32] Nakamura S., Suzuki H. & Kanazawa T. The ATP-induced change of tryptophan fluorescence reflects a conformational change upon formation of ADP-sensitive phosphoenzyme in the sarcoplasmic reticulum Ca^2+^-ATPase. Stopped-flow spectrofluorometry and continuous flow-rapid quenching method. J. Biol. Chem. 269, 16015–16019 (1994). 8206898

[b33] Barrabin H., Scofano H. M. & Inesi G. Adenosinetriphosphatase site stoichiometry in sarcoplasmic reticulum vesicles and purified enzyme. Biochemistry 23, 1542–1548 (1984). 6232947 10.1021/bi00302a031

[b34] Weber K. & Osborn M. The reliability of molecular weight determinations by dodecyl sulfate-polyacrylamide gel electrophoresis. J. Biol. Chem. 244, 4406–4412 (1969). 5806584

[b35] Daiho T., Suzuki H., Yamasaki K., Saino T. & Kanazawa T. Mutations of Arg^198^ in sarcoplasmic reticulum Ca^2+^-ATPase cause inhibition of hydrolysis of the phosphoenzyme intermediate formed from inorganic phosphate. FEBS Lett. 444, 54–58 (1999). 10037147 10.1016/s0014-5793(99)00027-7

[b36] Laemmli U. K. Cleavage of structural proteins during the assembly of the head of bacteriophage T4. Nature 227, 680–685 (1970). 5432063 10.1038/227680a0

[b37] Lowry O. H., Rosebrough N. J., Farr A. L. & Randall R. J. Protein measurement with the folin phenol reagent. J. Biol. Chem. 193, 265–275 (1951). 14907713

[b38] Humphrey W., Dalke A. & Schulten K. VMD: visual molecular dynamics. J. Mol. Graph. 14, 33–38 (1996). 8744570 10.1016/0263-7855(96)00018-5

[b39] Juul B. . Do transmembrane segments in proteolyzed sarcoplasmic reticulum Ca^2+^-ATPase retain their functional Ca^2+^ binding properties after removal of cytoplasmic fragments by proteinase K? J. Biol. Chem. 270, 20123–20134 (1995). 7650031 10.1074/jbc.270.34.20123

[b40] Brandl C. J., Green N. M., Korczak B. & MacLennan D. H. Two Ca^2+^ ATPase genes: homologies and mechanistic implications of deduced amino acid sequences. Cell 44, 597–607 (1986). 2936465 10.1016/0092-8674(86)90269-2

